# The protective effect of breastfeeding on infant inflammation: a mediation analysis of the plasma lipidome and metabolome

**DOI:** 10.1186/s12916-025-04343-0

**Published:** 2025-10-08

**Authors:** Satvika Burugupalli, Toby Mansell, Tingting Wang, Alexandra D. George, Sudip Paul, Richard Saffery, Mimi L. K. Tang, Thomas W. McDade, Habtamu B. Beyene, Thy Duong, Peter Vuillermin, Anne-Louise Ponsonby, Barwon Infant Study Investigator Group, Fiona Collier, Lawrence Gray, Martin O’Hely, Sarath Ranganathan, Peter Sly, David P. Burgner, Peter J. Meikle

**Affiliations:** 1 Metabolomics Laboratory, Baker Heart and Diabetes Institute, 75 Commercial Road, Melbourne, VIC 3004 Australia; 2 Baker Department of Cardiometabolic Health, University of Melbourne, Parkville, Australia; 3 Murdoch Children’s Research Institute, Royal Children’s Hospital, 50 Flemington Road, Parkville, VIC 3052 Australia; 4 Department of Paediatrics, University of Melbourne, Parkville, Australia; 5 Department of Cardiovascular Research, Translation and Implementation, La Trobe University, Bundoora, Australia; 6 Department of Anthropology and Institute for Policy Research, Northwestern University, Evanston, IL USA; 7 School of Medicine, Deakin University, Geelong, Australia; 8 Barwon Health, Geelong, Australia; 9 Florey Institute of Neuroscience and Mental Health, University of Melbourne, Melbourne, Australia

**Keywords:** Breastfeeding, Lipids, Metabolites, Infant nutrition, Inflammation

## Abstract

**Background:**

Inflammation has long-term health impacts across the life course. Breastfeeding substantially reduces inflammation risk, but key pathways, including the extent that this is due to protection against early life infection, are poorly understood. We aimed to investigate the relationships between breastfeeding, inflammation, and infection burden, and to determine the extent to which metabolomic and lipidomic profiles associated with breastfeeding mediate these health outcomes.

**Methods:**

We utilised data from the Barwon Infant Study (BIS), a longitudinal birth cohort in Victoria, Australia. Infants (*n* = 889) with available breastfeeding (categorised as yes/no) clinical, metabolomic, and Lipidomic data at 6 and/or 12 months were included (*n* = 793 at 6 months, *n* = 734 at 12 months). Inflammation, measured via glycoprotein acetyls (GlycA), at 6 and 12 months and infection burden, including parent-reported and medically attended infections assessed through standardised 3-monthly questionnaires were used as outcomes.

**Results:**

Any breastfeeding, regardless of supplementary feeding, was associated with lower inflammation, fewer infections, and significant, potentially beneficial changes in metabolomic and lipidomic markers, particularly plasmalogens. There was evidence of bidirectional mediation: metabolomic biomarkers and lipids mediated breastfeeding’s effects on inflammation, while inflammation partly mediated breastfeeding’s impact on certain metabolites and lipids.

**Conclusions:**

These findings highlight pathways through which breastfeeding reduces inflammation and infection burden, identifying potential targets for optimising infant feeding.

**Supplementary Information:**

The online version contains supplementary material available at 10.1186/s12916-025-04343-0.

## Background

The beneficial effects of breastfeeding on infant and maternal health outcomes are well-recognised, and the World Health Organization (WHO) recommends breastfeeding for optimal early life nutrition [1]. In infancy, breastfeeding is associated with lower infection burden and severity [2, 3] and a reduced risk of childhood non-communicable diseases (NCDs) and adverse conditions, including asthma [4] and obesity [5]. There is also evidence for longer-term benefits, including reduced risk of later cardiovascular disease (CVD) [6, 7], obesity, and type 2 diabetes [7, 8]. The mechanisms underlying these epidemiological observations are poorly understood but may include beneficial effects on immune function and reduced inflammation in early life. 

Inflammation is an essential and evolutionarily conserved response to infection and injury, but dysregulated, poorly resolving inflammation has adverse effects across the life course. In adults, disruption of immune homeostasis leading to dysregulated inflammatory responses is a key and potentially modifiable pathogenic mechanism in both communicable diseases and NCDs [9–12]. Levels of inflammatory biomarkers and functional mediators correlate with the severity and outcomes of many infections [13, 14], and adjunctive anti-inflammatory interventions (in addition to antimicrobials) improve outcomes in some instances [15, 16]. Chronic inflammation predicts CVD events, metabolic disease, cancer, dementia, respiratory disease, and overall mortality, and is an emerging therapeutic target in adult-onset NCDs [17–19].

There are fewer analogous data on inflammation in infancy and childhood. The initial pathogenic changes of many NCDs are suggested to begin early in life [20], and are likely partly driven by inflammation [21]. Therefore, reducing adverse inflammation earlier in life may benefit lifelong health [22]. Relatively little is known about the drivers of early life inflammation. Infection and microbial exposures are potent inflammatory stimuli [23], and we have previously found evidence for higher infection burden associating with higher inflammation in infancy [24], measured by glycoprotein acetyls (GlycA) and high-sensitivity C-Reactive Protein (hsCRP). GlycA and hsCRP are suggested to capture distinct but overlapping inflammatory pathways [25]. In contrast to hsCRP, a marker of acute inflammation particularly in childhood, GlycA better captures chronic inflammation burden over time [13] especially in early life [26]. Breastfeeding may impact lipid metabolism [27] and infection burden [24], thereby influencing overall inflammation directly and indirectly through effects on immune regulation. 

**Fig. 1:**
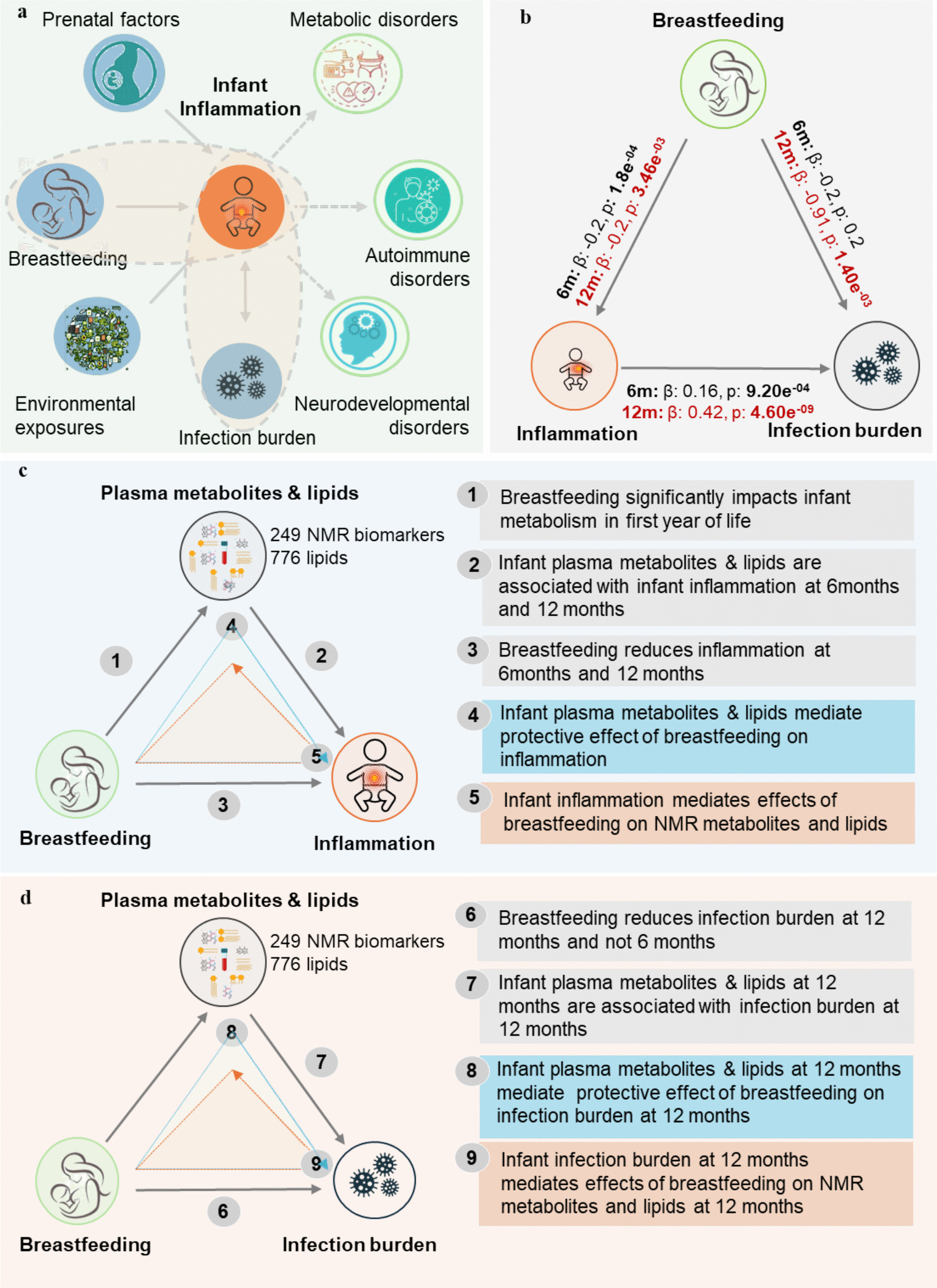
Graphical study abstract.** a** Antenatal and postnatal factors that increase childhood inflammation and consequences of chronic inflammation. **b** Interaction between breastfeeding, infection, and inflammation. **c** Main findings of the effects of breastfeeding, inflammation, and metabolism and mediating pathways. **d** Main findings of the effects of breastfeeding, infection burden, and metabolism and mediating pathways

To address these knowledge gaps and explore underlying mechanisms, we investigated the associations between breastfeeding, chronic inflammation, and infection burden at 6 and 12 months of age. We also examined the extent and direction of mediation of these relationships; in particular, whether plasma Lipids and other metabolites at 6 and 12 months of age mediated the protective effect of breastfeeding on inflammation and infection burden, and conversely, whether reduced inflammation and infections mediated the effects of breastfeeding on plasma lipids and metabolites (see Fig. 1 for graphical abstract).

## Methods

A summary of the analyses and the corresponding tables is shown in Additional file 1: Table S1.

### Study cohort

We used data from the Barwon Infant Study (BIS), a pre-birth, population-derived longitudinal cohort with an unselected, antenatal sampling frame based in the southeast of Australia. BIS was established to investigate the biological processes mediating the effect of prenatal and early life environmental exposures on NCD risk, with a focus on immune, respiratory, cardiovascular, and neurodevelopment domains [28]. Recruitment for BIS took place from 2010 to 2013. The inclusion and exclusion criteria for BIS have been detailed previously [28]. In brief, mothers were recruited before 32 completed weeks of pregnancy and were eligible if they were residents of the Barwon region and planned to give birth at the local hospitals. Exclusion criteria for mothers included age < 18 years, not being a permanent Australian resident, or need for an interpreter to complete questionnaires. Exclusion criteria for infants included being born very preterm (< 32 completed weeks gestation) or major congenital malformations or serious illness were identified within the first few days of Life. For this study, all BIS participants with available breastfeeding data, metabolomic and Lipidomics data at 6 months and/or 12 months of age were included (*n* = 793 at 6 months, *n* = 734 at 12 months). A participant flow chart is shown in Additional file 1:Fig. S1. Mothers provided written informed consent at recruitment, and study ethics was approved by the Barwon Health Human Research Ethics Committee (HREC 10/24).

### Breastfeeding and parent-reported infant infection data

Mothers reported on breastfeeding and infant infections in questionnaires collected at 1 month, 3 months, 6 months, 12 months, and 18 months post-birth. For this study, the breastfeeding exposures of interest were current breastfeeding at the 6-month time point and at the 12-month time point (binary yes/no at each time point), defined as duration of any breastfeeding of at least 26 weeks and at least 52 weeks, respectively. Sixty-one percent (487/793) and 39% (384/734) of participants were breastfed at 6 and 12 months, respectively. Most participants WHO were breastfed were fed directly, with only 6% (40/793) and 1% (9/734) of participants receiving any expressed breast milk at 6 and 12 months, respectively.

Parent-reported infection burden was defined as the total number of infant respiratory tract infections, gastroenteritis, conjunctivitis, and acute otitis media episodes reported by mothers between birth and 6 months for 6-month analyses, and between birth and 12 months for 12-month analyses [29]. Attendance to a general practitioner (GP) due to infant infection was defined as the total number of parent-reported visits to a GP due to infant infection over the same periods of time. Parents reported a median of 2 (interquartile range, IQR 1–3) child infections in the first 6 months of Life and 5 total infections (IQR 3–8) in the first year of life (Additional file 1: Table S2). Infants attended a general practitioner (GP) due to infection a median of once (IQR 1–3) in the first year of Life. Distributions in this cohort of infections, infection-related health care utilisation, and risk factors for infections such as childcare attendance have been reported previously. Approximately two-thirds of infants had not attended childcare by 12 months of age [30]. The majority of infants (> 91%) in the geographical region of BIS were fully vaccinated in line with the Australian vaccination schedule [31].

### Maternal and infant covariates

Potential confounders were selected for model adjustment a priori, consistent with previous studies using these metabolomic and lipidomic data [24, 27, 32]. Self-reported maternal age (years), pre-pregnancy body mass index (BMI) (kg/m^2^), smoking during pregnancy (dichotomised here as any or none), education (dichotomised as completed any university level certificate or less than university level education), and infant ethnicity (dichotomised as Anglo/European or ethnic minority based on parental country of birth, due to small numbers in original country groupings [33]) were collected from questionnaires. Infant gestational age (weeks) and birth weight (kg) were collected from hospital reports. Age- and sex-standardised birth weight z-score was calculated from the 2009 revised United Kingdom WHO growth charts [34]. Area-level socioeconomic disadvantage (Index of Relative Socio-Economic Disadvantage) based on the 2011 Socio-Economic Indexes for Areas (SEIFA) was derived by mapping participants’ addresses to the statistical areas used in the index [35]. In sensitivity analyses, infant weight at 6 months and 12 months measured by research staff with a digital scale (Seca Digital Baby Scale 354) was used as a potential confounder [36].

### GlycA and other NMR metabolomics data

Nuclear magnetic resonance (NMR) metabolomics was performed on infant plasma collected at the 6-month and 12-month time points. This data has been published previously [24]. Briefly, venous peripheral blood was collected in sodium heparin and generally processed within 4 h of collection. Plasma was stored at − 80 °C until shipment on dry ice to Nightingale Health (Helsinki, Finland) for NMR metabolomic measurement. The NMR metabolomic platform quantifies GlycA and a broad range of lipoprotein and other metabolite biomarkers, including cholesterols, triglycerides, fatty acids, amino acids, and apolipoproteins. The platform has been described in detail previously [37, 38] and was initially used to quantify 228 biomarkers in the 12-month samples using the 2016 Nightingale Health bioinformatics protocol. Subsequently, 250 biomarkers were quantified in the 6-month samples using the 2020 bioinformatics protocol (224 biomarkers common to both time points). The median (IQR) of GlycA at 6 and 12 months was 0.76 mmol/L (0.68–0.85) and 1.28 mmol/L (1.15–1.46), respectively (Additional file1: Table S2).

### Lipidomic data

The detailed methodology and data processing used in BIS lipidomics has been previously published [39]. In brief, 10 µL of plasma was mixed with 100 µL of butanol: methanol (1:1) with 10 mM ammonium formate containing a mixture of internal standards. Samples were vortexed, sonicated for an hour and then centrifuged (14,000 × g, 10 min, and 20 °C) before transferring the supernatant into sample vials with glass inserts for analysis [40]. Lipidomics was performed as described previously in [41] with adaptations for a dual column setup. Analysis of plasma extracts was performed on an Agilent 6490 QQQ mass spectrometer with an Agilent 1290 series HPLC system and two ZORBAX eclipse plus C18 column (2.1 × 100 mm 1.8 mm, Agilent) with the thermostat set at 45 °C. Mass spectrometry analysis was performed in both positive and negative ion mode with dynamic scheduled multiple reaction monitoring (MRM). Lipid class total concentrations were calculated as the sum of individual lipid species concentrations within the class, except in the case of triacylglycerol (TGs) and alkyl-diacylglycerol (TG (O)s), where we measured both neutral loss [NL] and single ion monitoring [SIM] peaks, and subsequently used the more accurate, but less structurally resolved, [SIM] species concentrations for summation purposes when examining lipid class totals.

### Statistical analyses

All statistical analyses were carried out in R (3.5.0 or 4.0.3). Plasma metabolomic biomarkers and lipid concentrations were log-10 transformed and scaled to a standard distribution (with mean of 0 and standard deviation of 1) prior to further statistical analysis. For descriptive summary of cohort characteristics (Additional file1: Table S2), mean and standard deviation or median and inter-quartile range were used for continuous variables, while number and percentage were used for binary variables. To describe the correlations between metabolomic and lipidomic measures, Spearman’s correlations (*ρ*) were calculated along with their corresponding two-sided *p*-value. To address missingness of model covariates (see below), multiple imputation using chained equations [42] was performed to generate 40 imputed datasets. Variables used for imputation were model covariates, breastfeeding status at both time points, number of infections at both time points, metabolomics and Lipidomics variables at both time points, and maternal height and weight at 28 weeks gestation. All models used the imputed datasets, with estimates across imputed datasets combined using Rubin’s rules [43].

#### Linear regression analyses

Associations between breastfeeding status, infection burden, and inflammation (GlycA) were assessed using univariate linear regression models. Associations between infant infection burden or GlycA and metabolomic biomarkers/lipid species were investigated using multiple linear regression, with separate models for each biomarker/lipid [44]. All multiple linear regression models were adjusted for sex, age, ethnicity, birth weight, gestational age, maternal education level, maternal smoking during pregnancy, pre-pregnancy BMI, and area-level socioeconomic disadvantage. All p-values are two-sided and were corrected for multiple comparisons, per model, using the Benjamini–Hochberg method [45].

#### Causal mediation analyses

Four sets of causal mediation analyses (using the R package “mediation”) [46] were performed to investigate the mediation of the effect of breastfeeding on the following: (1) GlycA by metabolomic biomarkers and lipid species; (2) metabolomic biomarkers and lipid species by GlycA; (3) infection burden by metabolomic biomarkers and lipid species; (4) metabolomic biomarkers and Lipid species by infection burden. As there was Limited evidence for longitudinal associations between breastfeeding status at 6 months or 6-month metabolomic biomarkers and Lipids with 12-month GlycA, cross-sectional mediation analyses at each time point (6 months and 12 months) were performed, using breastfeeding status, metabolomic biomarkers and lipids, GlycA, and total infection burden from the same time point.

To assess the extent to which the metabolomic biomarkers/lipids mediate the effects of breastfeeding on GlycA, we started with estimating the total effects of breastfeeding on GlycA using linear regression, adjusted for sex, age, ethnicity, birth weight, gestational age, maternal education level, maternal smoking during pregnancy, pre-pregnancy BMI, and area-level socioeconomic disadvantage. Next, we constructed the mediator model to examine the associations of breastfeeding with metabolomic biomarkers/lipid species, adjusted for the same covariates. Subsequently, we constructed a causal mediation model to estimate the proportion of effects explained by a direct effect of breastfeeding on GlycA—the average direct effect (ADE)—and the proportion that was mediated by metabolomic biomarkers/lipid—the average causal mediation effect (ACME). Confidence intervals and two-sided p-values were estimated empirically using 10,000 bootstraps resampling. We followed the same approach for the other mediation models (2–4 above). All causal mediation analyses were performed on data from the 6-month (*n* = 793 for models with GlycA, *n* = 577 for models with infection burden) and 12-month time points (*n* = 734 for GlycA, *n* = 549 for infection burden) separately.

As a sensitivity analysis for the assumption in the main analyses that infant weight was causally influenced by breastfeeding status, and as such was not a potential confounder of any effects of breastfeeding on metabolomic biomarkers, lipids, or GlycA, we repeated mediation analyses additionally adjusting for infant weight at the relevant time point (6 months or 12 months) as a confounder.

### Data representation

**Fig. 2:**
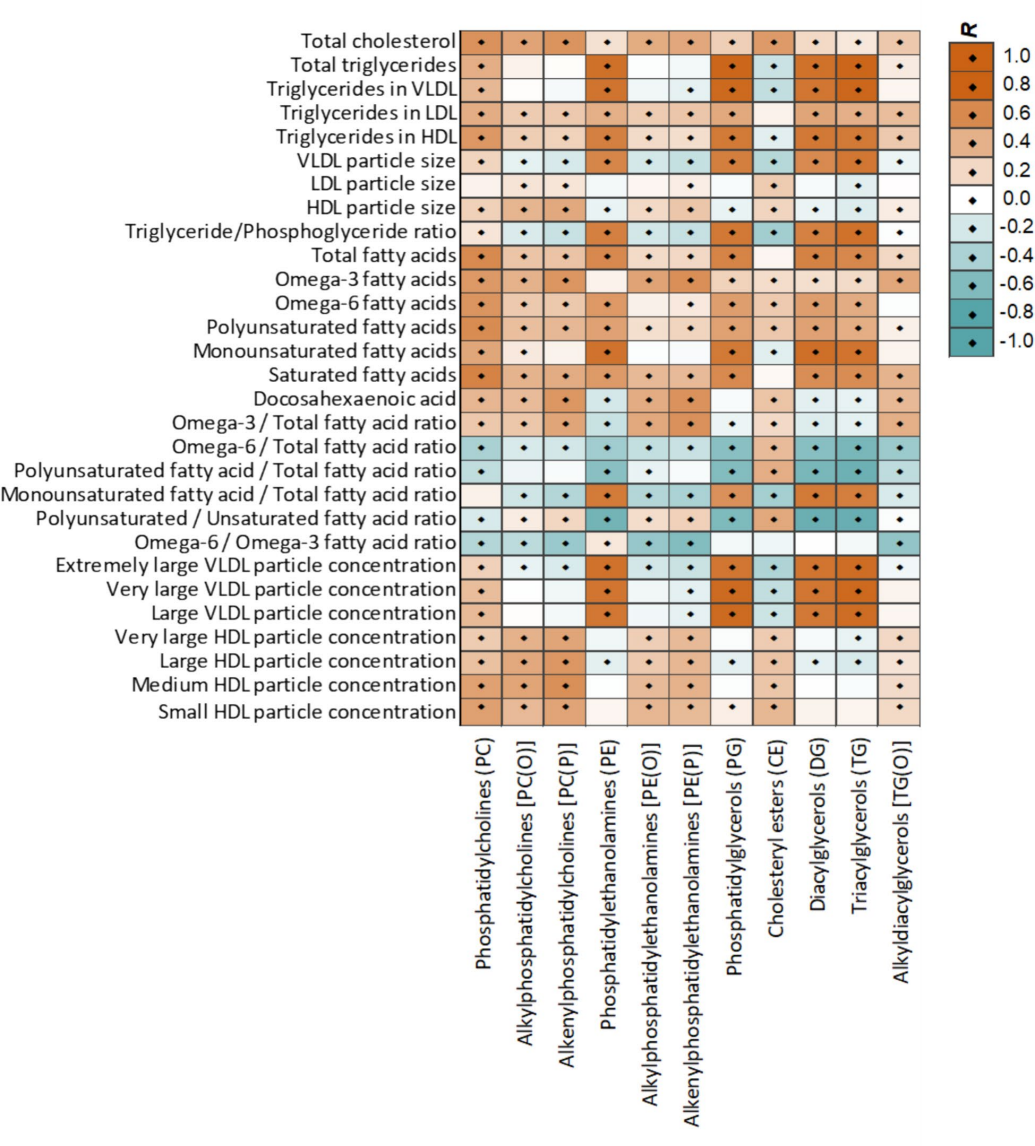
Spearman’s rank correlations between subsets of metabolomic biomarkers and Lipid classes at 6 months of age. Diamonds indicate p-values (*p* < 0.05) for correlations. Correlations for all the metabolomic biomarkers and Lipid species at 6 months and 12 months are shown in Additional file 1: Table S5 and Additional file 1: Table S6, respectively

**Fig. 3:**
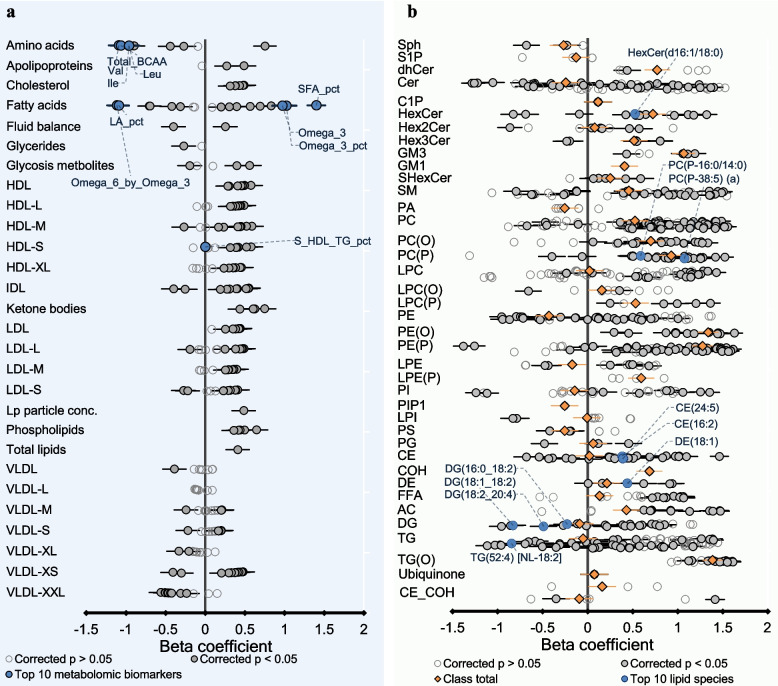
Association of breastfeeding status with metabolomic biomarkers and Lipids at 6 months. The forest plots represent the association between **a** metabolomic biomarkers and **b** plasma Lipids and breastfeeding at 6 months of age. Beta coefficient represents the mean difference of each metabolomic biomarker/lipid species in infants who are currently breastfed compared to those who are not, estimated using linear regression models adjusted for breastfeeding, sex, age, ethnicity, birth weight, gestational age, maternal education level, maternal smoking during pregnancy, pre-pregnancy BMI, and area-level socioeconomic disadvantage. Each circle on the plot represents a metabolomic biomarker/lipid species: open circles show biomarkers/lipid species with adjusted *p* > 0.05, grey closed circles show biomarkers/lipid species with adjusted *p* < 0.05, biomarkers/lipid species with the smallest *p-*values (top 10) are represented as closed blue circles. Orange diamonds represent Lipid class totals calculated as the sum of Lipid species in that particular lipid class. Bars represent 95% confidence intervals. Class membership of metabolomic biomarkers and Lipids is Listed in Additional file 1: Tables S3 and S4

**Fig. 4:**
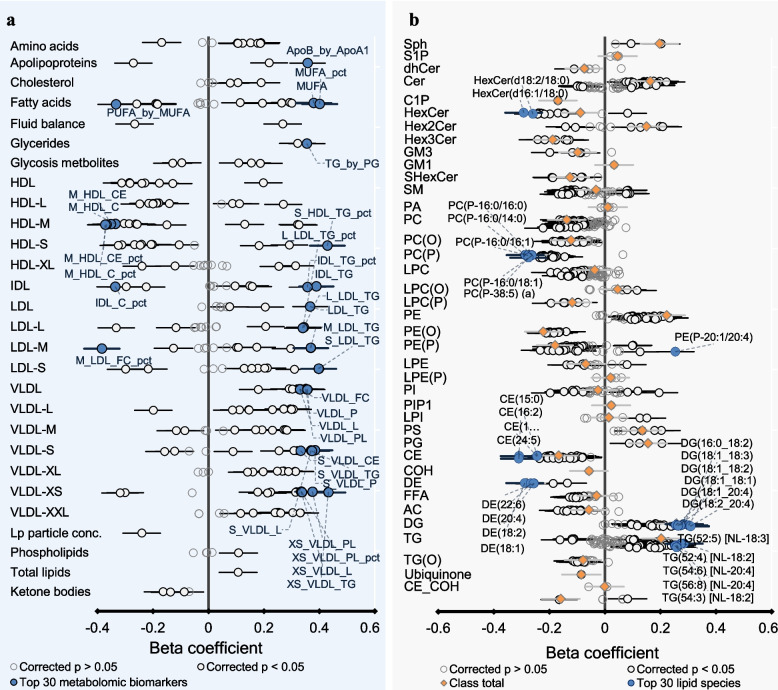
Association of metabolomic markers and Lipids with GlycA at 6 months. The forest plots represent the association between **a** metabolomic biomarkers and inflammation and **b** plasma lipids and inflammation. Beta coefficient represents the mean difference in standard deviation units of each metabolomic biomarker/lipid species on the log-10 scale per standard deviation change in GlycA, estimated using linear regression models adjusted for breastfeeding, sex, age, ethnicity, birth weight, gestational age, maternal education level, maternal smoking during pregnancy, pre-pregnancy BMI, and area-level socioeconomic disadvantage. Each circle on the plot represents a metabolomic biomarker/lipid species: open circles show biomarkers/lipid species with adjusted *p *>0.05, white closed circles show biomarkers/lipid species with adjusted *p *<0.05, biomarkers/lipid species with smallest *p*-values (top 30) are represented as closed blue circles. Orange diamonds represent Lipid class totals calculated as the sum of Lipid species in that particular lipid class. Bars represent 95% confidence intervals. Class membership of metabolomic biomarkers and Lipids is Listed in Additional file 1: Tables S3 and S4

**Fig. 5:**
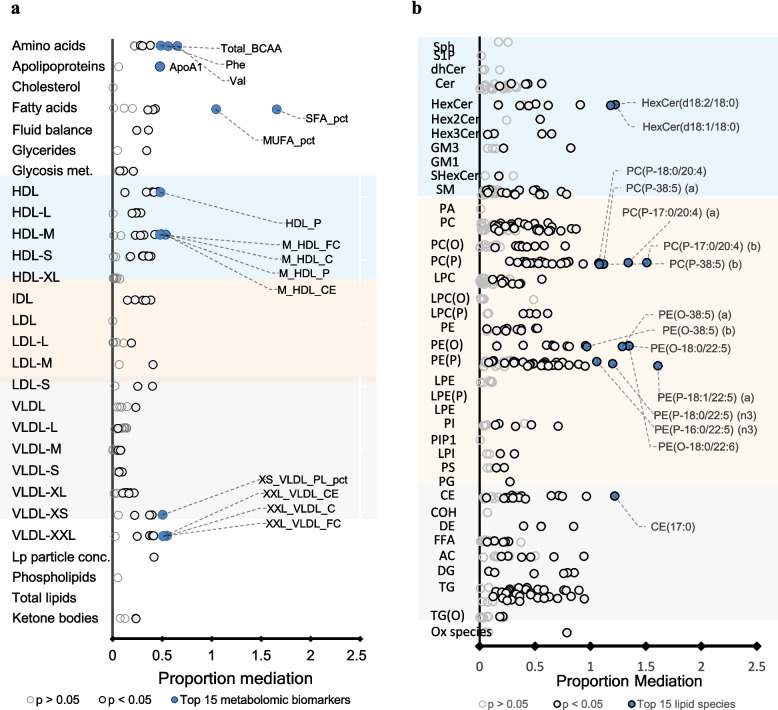
Metabolomic biomarkers and lipids mediate the protective effect of breastfeeding on GlycA. The forest plots represent the proportion mediation by **a** metabolomic biomarkers and **b** plasma lipids. Proportion mediation was estimated using mediation models to calculate the average causal mediation effect (ACME) mediated by the biomarker/lipid divided by the total effect of breastfeeding status on GlycA, adjusted for sex, age, ethnicity, birth weight, gestational age, maternal education level, maternal smoking during pregnancy, pre-pregnancy BMI, and area-level socioeconomic disadvantage. Each circle on the plot represents a metabolomic biomarker/lipid species: open circles show biomarkers/lipid species with *p* > 0.05, white closed circles show biomarkers/lipid species with *p* < 0.05, and biomarkers/lipid species with the highest proportion mediation (top 15) are represented as closed blue circles. Class membership of metabolomic biomarkers and Lipids is Listed in Additional file 1: Tables S3 and S4

The results are presented as a heat map in Fig. 2 and forest plots in Figs. 3, 4, and 5 where each metabolomic biomarker/lipid species (or class) is represented by a round marker showing the beta coefficient from linear regression models (Figs. 3 and 4) or proportion mediation from causal mediation analyses (Fig. 5). The colour of the markers relates to the magnitude of the associated *p*-value as indicated in the figure legends. The lipid species are arranged according to the pathway relatedness.

## Results

### Associations between breastfeeding, GlycA, and infections at 6 months and 12 months of age

We estimated associations between breastfeeding, inflammation, and parent-reported infections in participants from the Barwon Infant Study (BIS, a population-derived pre-birth cohort from the Barwon region of Victoria, Australia) [28] with available breastfeeding, clinical, metabolomic, and Lipidomic data at 6 months and/or 12 months of age (*n* = 899, 51% male). Participant characteristics are shown in Additional file 1: Table S2.

In univariate Linear regression analyses, breastfeeding at 6 months was negatively associated with GlycA levels at 6 months (*β* −0.2, *p* < 0.0001) but not with GlycA levels at 12 months (*β* 0.00, *p* = 0.98). Breastfeeding at 6 months was not associated with infection burden (number of parent-reported infections) by 6 months (*β* −0.12, *p* = 0.42) or infection burden by 12 months (*β* −0.26 *p* = 0.34). Breastfeeding at 12 months was negatively associated with GlycA levels at 12 months (*β* −0.04, *p* = 0.02) and with lower infection burden by 12 months (*β* −0.80, *p* = 0.003). A greater number of infections was associated with higher GlycA at both time points (6 months: *β* 0.01, *p* = 0.005; 12 months: *β* 0.01 *p* < 0.0001) (Fig. 1b).

Breastfeeding at 12 months was associated with lower GP attendances due to infection by 12 months (*β* −0.41, *p* = 0.001), and a higher number of these attendances was modestly associated with higher GlycA at both time points (6 months: *β* 0.02, *p* = 0.01; 12 months: *β* 0.01, *p* = 0.07). As parent-reported infections are a more comprehensive measure of infection burden than GP attendance and less biased by factors such as healthcare access and health literacy [47], the total number of parent-reported infections was used as the measure of infection burden for subsequent analyses.

### Correlation between metabolomic biomarkers and plasma lipids

GlycA and other metabolomic biomarkers were quantified by NMR metabolomics (250 biomarkers at 6 postnatal months, 228 biomarkers at 12 months) [24]. Lipid species were measured using a high-performance liquid chromatography/mass spectrometry (LC/MS) platform (776 lipid species at both time points) as described previously [39]. The NMR metabolomics platform provides a comprehensive profile of lipoprotein distribution and composition, fatty acid profile and other polar metabolites. The fatty acid profile measured by NMR includes total fatty acids, omega-3 and omega-6 fatty acids, polyunsaturated and monounsaturated fatty acids, as well as several ratios relative to total fatty acids (Listed in Additional file 1: Table S3), and the Lipidomics platform measures Lipid species spanning across 39 lipid classes (Additional file 1: Table S4). Together, these complementary platforms allow a detailed assessment of the metabolic pathways that may be influenced by infant feeding status.

At 6 months, all 250 metabolomic biomarkers were positively correlated with at least one of the 39 Lipid classes, and 197 biomarkers were also negatively correlated (at *p* < 0.05) with at least one Lipid class. At 12 months, 227 (of 228) metabolomic biomarkers were positively correlated, and 191 were negatively correlated with at least one lipid class. Correlations between metabolomic biomarkers and lipid class totals are shown in Additional file 1: Table S5 (6 months) and Additional file 1: Table S6 (12 months).

Lipid classes of phosphatidylethanolamine (PE), phosphatidylglycerol (PG), di- and tri-acylglycerols (DG, TG) showed strong positive correlations with very low-density (VLDL) particle size, TG/PG ratio, and the proportion of extremely large (XXL) to large (L) VLDL particles. These lipid classes were negatively correlated with low-density lipoprotein (LDL) and high-density lipoprotein (HDL) particle size, as well as the proportions of XL, L, and medium (M) HDL particles. In contrast, ether lipid classes such as alkyl phosphatidylcholine (PC (O)), alkyl phosphatidylethanolamine (PE (O)), alkenyl phosphatidylcholine (PC (P)), alkenyl phosphatidylethanolamine (PE (P)), alkyl-diacylglycerol (TG (O)) and cholesteryl esters (CE) showed positive correlations with HDL size and XL- to M-HDL lipoprotein particles and negative correlations with VLDL lipoprotein particle size, TG/PG ratio, and the proportion of XXL- to L-VLDL particles (Fig. 2).

Most of these lipid classes (PE, PG, DG, TG, PC (O), PE (O), PC (P), PE (P), and TG (O)) were positively correlated with total fatty acids, omega-3, omega-6, and polyunsaturated fatty acids (PUFA). Lipid classes of PE, PG, DG, and TG were negatively correlated with docosahexaenoic acid (DHA), the ratio of omega-3 to total fatty acids, and PUFA/monounsaturated fatty acids (MUFA) ratio, and positively correlated with MUFA. PC (O), PC (P), PE (P), PE (O), CE, TG (O), and PC were positively correlated with DHA, the ratio of omega-3 to total FA, and PUFA/MUFA ratio, and negatively correlated with MUFA. Cholesterol esters were positively correlated with ratios of omega-6 and PUFA to total fatty acids (Fig. 2).

### Breastfeeding is associated with specific infant plasma metabolomic and lipid profiles

We performed Linear regression modelling to investigate the cross-sectional associations of breastfeeding status with each metabolomic biomarker and Lipid at 6 and 12 months. Models were adjusted for sex, age, ethnicity, birth weight, gestational age, maternal education level, maternal smoking during pregnancy, pre-pregnancy BMI, and area-level socioeconomic disadvantage as potential pre-specified confounders (see Methods for details).

At 6 months, breastfeeding status was associated with 66% (165/250) of metabolomic biomarkers and 89% (656/733) Lipids at a 5% false discovery rate (FDR) **(**Fig. 3, Additional file 1: Tables S7–S9). Some of the strongest positive associations were seen with omega-3 fatty acids, ratios of omega-3 and saturated fatty acids (SFA) to total fatty acids, glutamine, and β-hydroxybutyrate. Breastfeeding was negatively associated with branched-chain amino acids (leucine, isoleucine, valine), tyrosine, phenylalanine, and omega-6 to omega-3 fatty acid ratio. Breastfeeding was positively associated with total HDL lipoproteins carrying lipids but negatively associated with XXL-VLDL lipoproteins.

Similar to previous findings in this cohort [39], breastfeeding was strongly positively associated with alkyl and alkenylphospholipids (PC (O), PE (O), PC (P), PE (P)), and alkyldiacylglycerols (TG (O)) lipid species. Breastfeeding was positively associated with several lipid species containing odd- and branched-chain fatty acids, including phosphatidylcholine (PC) (33:0), sphingomyelin (SM) (d19:1/24:1). Three lipid species characterised by an odd/branched chain fatty alcohol at sn-1, PE (P-15:0/22:6) (a), PE (P-15:0/20:4) (a) and PC (P-15:0/20:4) (a), showed a negative association with breastfeeding.

At 12 months, breastfeeding status was associated with 14% (32/228) of metabolomic biomarkers and 56% (411/733) of lipids (Additional file 1: Tables S10–S12), with **s**imilar patterns of associations to those observed at 6 months, albeit with smaller effect size estimates and wider confidence intervals compared to 6 months.

At both 6 (Additional file 1:Tables S13–S15) and 12 (Additional file 1: Tables S16–S18) months, lipid classes, including ceramides, diacylglycerols (DG), triacylglycerols (TG), and phosphatidylethanolamine (PE) species, were positively associated with GlycA. In contrast, lipid classes such as hexosylceramide (HexCer), phosphatidylcholine (PC), ether lipid classes such as alkenyl phosphatidylethanolamine (PE (P)) and alkenylphosphatidylcholine (PC (P)), alkyl phosphatidylethanolamine (PE (O)), alkyl phosphatidylcholine (PC (O)), and cholesteryl esters (CE) were negatively associated with GlycA. Most PUFA-containing di- and triacylglycerols were positively associated with GlycA, while PUFA-carrying ether Lipids were negatively associated, except for 20:4-carrying PE (P) species (PE (P-20:1/20:4)).

In longitudinal models, 72 metabolomic biomarkers and 104 Lipid species at 6 months were associated with GlycA at 12 months at the nominal *p* < 0.05 level; however, none were significant at the 5% FDR level (Additional file 1: Tables S19–S21).

### Plasma metabolomic biomarkers and lipids mediate the effects of breastfeeding on GlycA

Having established the associations between breastfeeding, GlycA, and metabolomic biomarkers and lipids, we performed mediation analysis to investigate the extent to which breastfeeding’s effect on lower GlycA was mediated through specific metabolic changes. Given the Limited evidence for longitudinal associations between both breastfeeding at 6 months and GlycA levels at 12 months, and between metabolomic biomarkers/lipids at 6 months and GlycA at 12 months, only cross-sectional mediation analyses were conducted at each respective time point (Additional file 1: Tables S22-S30).

At 6 months, 82 metabolomic biomarkers and 241 lipid species were estimated to mediate the effect of breastfeeding on GlycA (proportion mediated (M) 0.05–1.66, median 0.36, IQR 0.24 to 0.46) at a *p* < 0.05 level (Fig. 5, Additional file 1: Tables S22 and S23). At 12 months, 40 metabolomic biomarkers and 166 lipid species were estimated to mediate the effect of breastfeeding on GlycA (M 0.09–0.58, median 0.33, IQR 0.22 to 0.45) (Additional file 1: Tables S24 and S25).

The metabolomic biomarkers with the strongest evidence for mediating the effect of breastfeeding on GlycA at 6 months of age included the ratio of MUFA to total fatty acids, valine and phenylalanine, HDL-C, and HDL phospholipid (PL) measures. PUFA-containing alkenyl phosphatidylethanolamine, alkenyl phosphatidylcholine (PE (P), PC (P)), and alkyl phosphatidylethanolamine (PE (O)) species such as PE (P-18:0/22:5), PE (P-18:1/22:5), PC (P-17:0/20:4), PE (O-18:0/22:5), and PE (O-18:0/22:6) showed the highest proportion of mediation among lipid species (M 1.22–1.61). This proportion exceeding 100% suggests that the ACME via plasmalogens is larger than the total effect, which can occur when the direct and mediation effects act in opposite directions. In this case, breastfeeding is negatively associated with GlycA, positively associated with plasmalogens, and plasmalogens are negatively associated with GlycA. However, the positive association between breastfeeding and plasmalogens is stronger than the negative direct association between breastfeeding and inflammation, resulting in an ACME that exceeds the total effect. This pattern indicates that plasmalogens may play a dominant role in mediating the relationship between breastfeeding and systemic inflammation.

### Infant plasmalogen score mediates the effect of breastfeeding on GlycA

**Fig. 6:**
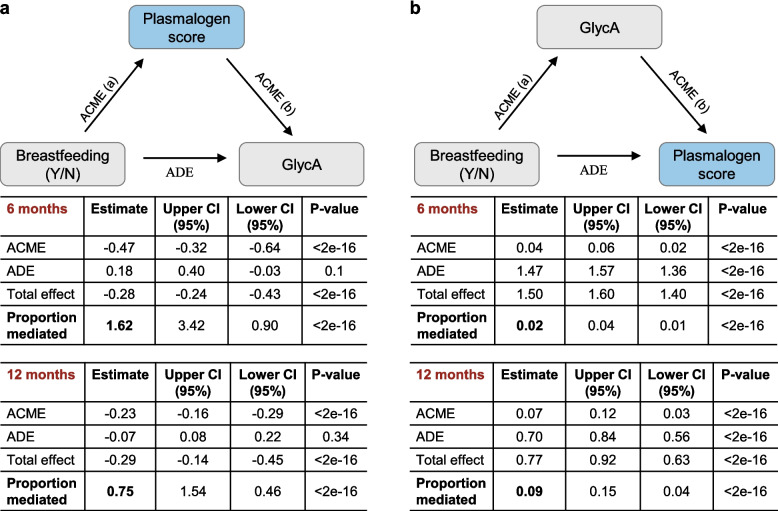
Mediation analyses of breastfeeding, plasmalogen score and GlycA. **a** Plasmalogen score as mediator of the effect of breastfeeding on GlycA. **b** GlycA as mediator of the effect of breastfeeding on plasmalogen score. Causal mediation analyses were performed, at both 6 months and 12 months, with breastfeeding as exposure. Average causal mediation effect (ACME) = product of the associations between the exposure and mediator, and the mediator and outcome; Total effect = average direct effect (ADE)+ACME; Proportion mediated = ACME/Total effect

As alkenyl phosphatidylethanolamine (PE (P)) species were positively associated with breastfeeding, negatively associated with GlycA, and mediated the protective effect of breastfeeding on GlycA, we next sought to explore this pathway in more detail. PE (P) levels have been reported to exist in a dynamic equilibrium with phosphatidylethanolamine (PE); to capture this, we have recently developed a plasmalogen score that is associated with a range of cardiometabolic outcomes, including type 2 diabetes and CVD [48]. At 6 months, the plasmalogen score was estimated to mediate 162% of the total effect (proportion mediated: 1.62, i.e. average causal mediation effect (ACME) to total effect ratio of 1.62, resulting in a percentage > 100%) of breastfeeding on GlycA. At 12 months, the plasmalogen score mediated an estimated 75% of the total effect of breastfeeding on GlycA (Fig. 6a). Conversely, there was little evidence at either age of GlycA mediating the effects of breastfeeding on the plasmalogen score (Fig. 6b).

### GlycA partly mediates the effect of breastfeeding on plasma metabolomic biomarkers and lipids

To investigate if the breastfeeding-associated reduction in inflammation alters infant metabolism, we considered an alternative mediation model with GlycA as the mediator. At 6 months, 81 metabolomic biomarkers (proportion mediation (*M*) 0.02–0.58) and 246 lipid species (*M* 0.01–0.36), and at 12 months, 44 metabolomic biomarkers (*M* 0.03–77.02) and 148 lipid species (*M* 0.03–0.81) were altered by the breastfeeding-associated reduction of GlycA at a *p* < 0.05 level.

At 6 months, the strongest mediation effect of breastfeeding mediated by GlycA was seen for XXL-XL VLDL particles carrying any lipids (L) and particles specifically carrying phospholipids (PL), any cholesterol (C), free cholesterol (FC), or triglycerides (TG), and small, medium, and large HDL particles carrying L, PL, C, CE, and FC. GlycA mediated the effect of breastfeeding on several tri- and diacylglycerol species and only a few DG ceramides, ether lipid species, and plasmalogens (PC (P), PE (P), and PE (O)) although there was no consistent fatty acid pattern (Additional file 1: Tables S26 and S27 for 6 months, Additional file 1: Tables S28 and S29 for 12 months). As shown in Fig. 6, the mediation by plasmalogen score on GlycA was much higher (*M* 1.62 at 6 months) than the mediation of GlycA on the plasmalogen score (M: 0.02). Comparisons of estimated mediation for metabolomic biomarkers and Lipids mediating the effect of breastfeeding on GlycA at 6 months of age and corresponding mediation for these biomarkers/lipids altered by reduction in GlycA are shown in Additional file 1: Fig. S1. A comparison of the estimated mediation for each direction of mediation at each time point is shown in Additional file 1: Table S30.

### Association of infant plasma lipids with infections and mediation analyses

Similar to previous findings [24], we found no associations between the total number of infections by 6 months of age with 6-month infant plasma metabolomic biomarkers or Lipids at the 5% FDR level. At 12 months, the total number of infections was associated with 40 of 228 (18%) metabolomic biomarkers and 40 of 776 (5%) of lipid species (Additional file 1: Tables S31–S33). TG-carrying lipoproteins such as HDL-TG and VLDL-TG were positively associated with infection burden, and medium- to extra-large-sized HDL carrying cholesterol and phospholipids were negatively associated. The ratio of MUFA to total fatty acids was positively associated with infection burden, whereas ratios of PUFA, omega-3, and omega-6 to total fatty acids were negatively associated. Omega-6 (20:3, 20:4, 22:4) carrying phosphatidylethanolamine (PE), DG, and TG Lipid species were associated positively with infection burden, whereas several Lipid species of desmosterol, cholesteryl ester, ether lipids, plasmalogens, and hexosylceramides carrying omega 3 fatty acids (22:6) were negatively associated.

At 12 months, 21 of 228 (9%) of metabolomic biomarkers and 40 of 776 (5%) Lipid species mediated the effect of 12-month breastfeeding status on reducing total infection burden by 12 months (proportion mediated (*M*) 0.06–0.42) at the *p* < 0.05 level (Additional file 1: Table S34 and S35). DHA-containing ether Lipids showed the highest proportion of mediation. Plasmalogen score at 12 months partly mediated the effect of breastfeeding on lowering infection count (*M* 0.38).

In the mediation models with total infection burden by 12 months as a mediator of the effects of 12-month breastfeeding status on 12-month metabolomic biomarkers, infections mediated effects on 68 metabolomic biomarkers and 60 lipid species at the *p* < 0.05 level (Additional file 1: Tables S36 and S37). The strongest evidence for mediation by infections was for GlycA (*M* 0.21), followed by various subclasses of triglycerides, MUFA and PUFA ratios, HDL cholesterol, apolipoprotein A1, and phenylalanine.

For the mediation models, postnatal weight (infant weight at the 6-month and 12-month time points) was not adjusted for as it was considered a potential mediator of the effects of breastfeeding on metabolomic biomarkers and lipids and inflammation. As a sensitivity analysis, we considered an alternative assumption where infant weight influenced whether infants were breastfed. We also performed mediation analyses and adjusted infant weight cross-sectionally to the relevant time point (6 or 12 months). Findings from these models were very similar to the main mediation models (Additional file 1: Tables S38–S45).

In summary, we have shown that breastfeeding is linked to lower levels of inflammation. Notably, breastfeeding was positively associated with HDL lipoproteins, which are protective against inflammation, but negatively associated with XXL-VLDL lipoproteins, which are pro-inflammatory. The effect of breastfeeding on lowering inflammation is mediated by plasmalogen levels, which show a strong negative association with inflammation and a positive association with breastfeeding. In addition, inflammation mediates some of the effects of breast-feeding on metabolomic biomarkers and lipids, albeit to a lesser extent. The beneficial effects of breastfeeding on early life infection were largely mediated by DHA-carrying ether lipids and plasmalogen score.

## Discussion

Breastfeeding and inflammation were both associated with several classes of circulating plasma metabolomic biomarkers and Lipids at 6 and 12 months. Breastfeeding was modestly associated with reduced infections by 12 months of age, but more markedly with lower inflammation both at 6 months and 12 months, suggesting that the anti-inflammatory effects of breastfeeding extend beyond reducing infection-associated acute inflammation alone and impact other pathways that result in chronic inflammation.

We found evidence that metabolic pathways mediate these relationships. Most notably, plasma metabolomic biomarkers and Lipids largely mediated the protective effect of breastfeeding on inflammation. To a lesser extent, we also observed the reverse effect where inflammation mediated the effect of breastfeeding on some plasma metabolomic biomarkers and Lipids. Infection burden by 6 months was only modestly associated with 6-month plasma metabolomic biomarkers and Lipids, with stronger associations evident for infection burden by 12 months. Similar to inflammation, we found evidence both for plasma metabolomic biomarkers and lipids partly mediating the effect of breastfeeding on infection burden and, to a lesser extent, infection burden mediating the effect of breastfeeding on some plasma metabolomic biomarkers and lipids. Previous findings in this cohort support mediation by inflammation (as measured with GlycA) of the effects of infection burden on metabolomic and lipidomic profile differences in infancy [24].

The observed associations between lipoprotein subclasses and specific lipid species are biologically plausible and align with known structural and functional characteristics of these particles. For example, triglycerides and phospholipids are typically enriched in VLDL particles, while ether lipids such as plasmalogens are often found in HDL fractions, reflecting their roles in lipid transport and antioxidant defence, respectively [49, 50]. While these patterns have been described in adult populations, our study extends this knowledge to early life, a period of unique metabolic demands and rapid developmental change. This is particularly relevant as we show that these lipoprotein–lipid associations are already evident in infancy, suggesting early establishment of metabolic pathways that may influence long-term cardiometabolic risk [51]. Importantly, the enrichment of anti-inflammatory lipids such as plasmalogens in HDL provides a potential mechanistic explanation for the protective associations observed between breast feeding and systemic inflammation (measured by GlycA). These findings not only underscore the role of lipoprotein composition in shaping inflammatory profiles but also suggest that early nutritional exposures may influence lipid-mediated pathways that are biologically relevant to disease risk trajectories [52, 53].

Notably, three key metabolomic biomarkers/lipid classes exhibited a distinctive pattern associated with breastfeeding and inflammation.

### Lipoprotein type and particle size

In adults, increased triglycerides (TG), decreased HDL-C, and increased LDL-C are well-established clinically applicable markers for obesity and cardiovascular disease [54, 55] and are each indicative of a pro-atherogenic state [56]. In infants, we observed that breastfeeding was associated with lower TG, HDL-C, and LDL-C. Lower TG and higher HDL-C were both associated with lower inflammation. HDL-C partly mediated the protective effect of breastfeeding on inflammation. HDL-PL also partially mediated the effect of breastfeeding on inflammation, and its functional role is discussed further below.

In addition, inflammation mediated the effect of breastfeeding on XXL-XL VLDL particles carrying any lipids and those carrying cholesterol, TG, or PL, TG/PG ratio, as well as some M-L HDL particles. Similarly, in lipidomic analysis, reduced inflammation also mediated the effect of breastfeeding on di- and triacylglycerol (DG, TG) lipid species. Although the proportion of mediation by GlycA on lipoprotein/lipid biomarkers was generally lower than the reverse mediation, certain lipoprotein biomarkers, such as the apolipoprotein B to apolipoprotein A1 ratio, showed comparable mediation estimates in both directions (0.45 with GlycA as mediator, 0.43 with ApoB/ApoA1 ratio as mediator). These findings support potential bi-directional mediation of effects of breastfeeding on infection risk, inflammation, and some lipoprotein metabolic pathways.

In general, formula-fed infants gain more weight in infancy than breastfed infants, increasing adipose tissue and inflammatory cells, and contributing to systemic inflammation [57]. Breastfed infants have higher brown adipose tissue activation [58], which in turn is associated with higher levels of HDL-C [59] and may contribute to reduced systemic inflammation in breastfed infants. Elevated levels of LDL and oxidised LDL induce pro-inflammatory responses in immune cells [60], whereas HDL is considered beneficial and is associated with cardioprotective effects. The composition and function of HDL particles are altered by systemic inflammation and autoimmune diseases [61–63]. Infection and inflammation also affect lipid metabolism through cytokine production stimulation [60]. More recently, SARS-CoV-2 infection has been associated with differences in amino acid and fatty acid plasma concentrations [64]. Anti-inflammatory treatments in inflammatory diseases such as rheumatoid arthritis and psoriasis affect lipid levels, particularly HDL, although the biological mechanisms are largely unknown [61]. Our observation of similar patterns in infants emphasises the importance of understanding lipoprotein and lipid dysregulation in early life and the potential impact of intervening in these early adverse changes.

### Fatty acids

**Fig. 7:**
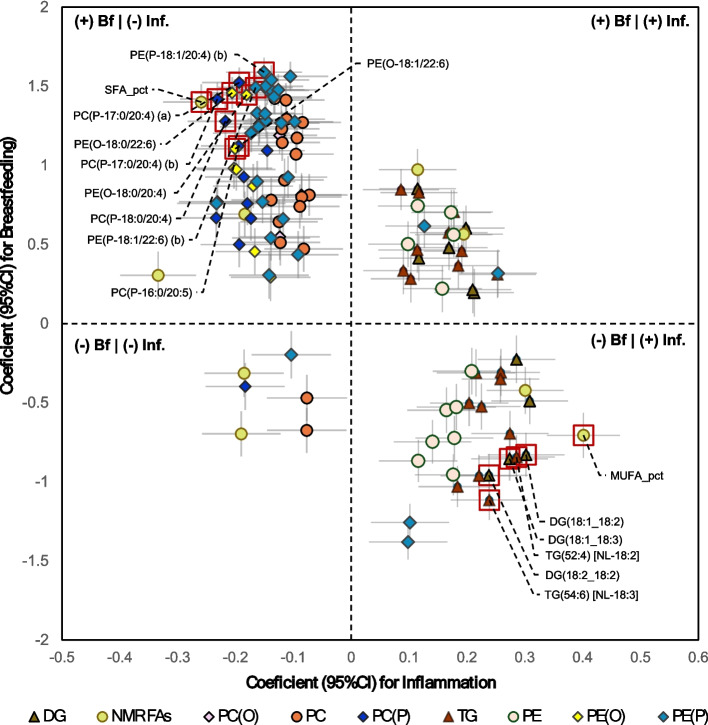
Summary of the cross-sectional associations at 6 months of total fatty acids, and selected PUFA-carrying lipid classes, with inflammation (GlycA, x-axis) and breastfeeding status (y-axis). Points are coefficients from adjusted Linear regression models. Red squares highlight mediators of the effect of breastfeeding on inflammation. Bars are 95% confidence intervals

Many metabolomic fatty acid biomarkers (ratios of SFA, MUFA, and DHA in total fatty acids, PUFA/MUFA ratio, and linoleic acid) showed evidence of mediation in both directions, i.e. these fatty acids mediated the effect of breastfeeding on inflammation, and inflammation mediated the effect of breastfeeding on these fatty acids, although generally to a lesser extent. For example, the proportion mediation of the effect of breastfeeding on inflammation at 6 months by linoleic acid (measured by the NMR metabolomic platform) is 0.43 (Additional file 1: Table S22) compared with 0.19 proportion mediation of the effect of breastfeeding on linoleic acid by inflammation (Additional file 1: Table S26). Total omega 3 fatty acids were positively associated with breastfeeding and inflammation at 6 months, while the ratio of DHA to total fatty acids was positively associated with breastfeeding and negatively associated with inflammation. However, neither omega-3 nor omega-6 fatty acids overall mediated the effect of breastfeeding on inflammation (Fig. 7).

It is well established that fatty acids play critical roles in inflammatory regulation and immune function [65, 66]. Fatty acids such as linoleic acid are integral to anti-inflammatory pathways and immune modulation [67, 68]. Conversely, infection and inflammation may regulate fatty acid synthesis, mobilisation, or metabolism [67]. This evidence for bidirectional mediation effects highlights the complex interplay between nutrition, lipid metabolism, and inflammation during early development, providing mechanistic insights into how breastfeeding may support optimal immune and metabolic health in infants.

### Ether lipids and plasmalogens

Ether lipids are characterised by an ether linkage on the glycerol backbone [69]. A key class of ether lipids, alkenyl phosphatidylethanolamines (PE (P)), commonly known as plasmalogens, have antioxidant properties due to their vinyl-ether bond, which scavenges reactive oxygen species (ROS) [70]. These lipids are also integral to immune cell structure and function [71].

Our findings showed that ether Lipids, particularly plasmalogens, were positively associated with breastfeeding and negatively associated with inflammation. Plasmalogens mediated a large protective effect of breastfeeding on inflammation as well as infection burden at 12 months. We also observed evidence for GlycA mediating some of the effect of breastfeeding on most of these ether Lipids at both time points, although the proportional mediation effect was much smaller; generally, less than 10% of the total effect.

We developed a plasmalogen score that encapsulates the relative circulating PE and PE (P) levels [48]. We show that breastfed infants have a higher plasmalogen score, which shows a proportion mediation of 1.62 in the protective effect of breastfeeding on inflammation at 6 months, in contrast to the reverse mediation pathway, where inflammation did not mediate the effect of breastfeeding on the plasmalogen score (estimated proportion mediation of 0.02). The unidirectional mediation by ether lipids/plasmalogen score supports the effect of breastfeeding on inflammation being largely driven by changes in these lipids.

We have previously shown that ether Lipids constitute approximately 0.45% of the total lipids in human milk but are absent in infant formula [72]. In breastfed infants, circulating ether Lipids such as alklydiacylglycerols are up to 16-fold higher than in formula-fed infants, with total PE (P) showing a 2–4-fold increase [73, 74], suggesting a role for breastfeeding-specific effects on infant lipid metabolism. The anti-inflammatory effects of plasmalogens could derive from their effects on the physical properties of immune cell membranes, which may influence cell signalling pathways and result in reduced inflammatory responses [48]. Plasmalogens also inhibit lipid peroxidation, a pathway that generates pro-inflammatory mediators [75]. Lipid peroxidation can also lead to ferroptosis, with T and B cells being particularly susceptible to this form of cell death [74]. Plasmalogens have been proposed to be anti-ferroptotic [76, 77], suggesting that reduced ferroptosis may be a mechanism underlying plasmalogens’ mediation of the relationship between breastfeeding and inflammation. Our findings, therefore, highlight the potential importance of plasmalogens in breast milk in modulating infant immune responses and reducing inflammation through multiple potential mechanisms.

In addition, we observed that several amino acids and related metabolites (including glutamine, total branched-chain amino acids, valine, leucine, isoleucine, tyrosine, and phenylalanine) were negatively associated with breastfeeding and, as previously reported [24], positively associated with inflammation. Glycine showed an inverse association pattern. Many of these amino acids showed evidence for mediating the effects of breastfeeding on inflammation. Infant formula has a higher protein content than breastmilk [78], but it is unknown if the proteins and subsequent amino acids in formula feeds have similar immunological effects to those in breastmilk.

In summary, we have shown that breastfeeding alters infant metabolism and reduces inflammation by modulating specific circulating metabolomic biomarkers and lipids. We demonstrate that HDL lipoproteins, ether lipids, and plasmalogens, particularly omega-3 carrying plasmalogens, are important mediators of the protective effect of breastfeeding on inflammation. Despite the proposed role of omega-3 PUFAs in infant immune development, we did not find evidence that omega-3 PUFA per se mediated the protective effect of breastfeeding on inflammation. Instead, our results indicate that the lipid carrier (plasmalogens) of omega-3 PUFA underpins the beneficial anti-inflammatory effects of breastfeeding. In addition, we found evidence that inflammation mediated the effects of breastfeeding on some metabolomic biomarkers and lipids, although this effect size was generally smaller and restricted to a different set of metabolomic biomarkers/lipids. Understanding the mechanisms underpinning the beneficial effects of breastfeeding, especially on early-life inflammation and infection burden, could inform interventions to optimise infant feeding, especially when breastfeeding is not possible or preferred. Our findings may also be relevant to high-risk infants, such as those born preterm. Mitigating the immediate and long-term effects of cumulative inflammation and adverse metabolomic and lipidomic profiles from early life may have pervasive effects on health outcomes across the life course [23] and consequentially reduce the longer-term risks of later onset diseases.

### Strengths and limitations

This study’s strengths include using two complementary omics platforms to measure Lipid and metabolomic biomarker profiles in a large prospective early Life cohort, and assessment of inflammation across two time points in infancy. Parent-reported infections were collected using standardised questionnaires at several time points from birth to 12 months, reducing measurement error due to the length of the recall period [29].

Limitations include the cross-sectional nature of the mediation analyses. Longitudinal analyses showed that breastfeeding at 6 months status was not associated with GlycA at 12 months, and there was Little evidence for longitudinal associations between 6-month metabolomic biomarkers and Lipids and 12-month GlycA. Consequently, we did not undertake analysis of mediation of longitudinal effects of breastfeeding across this time frame. Though breastfeeding status was measured at the same time as the outcomes, we consider it unlikely for there to be a substantial component of reverse causation with metabolomic biomarkers, lipids, inflammation, or infection influencing breastfeeding status. We acknowledge that while breastfeeding contributes significantly to the infant’s Lipid profile, particularly in early infancy, by 12 months of age infants are typically consuming a mixed diet. As such, the fatty acid composition measured in plasma at this time point may reflect broader dietary influences beyond breast milk, including the intake of solid foods. This could introduce variability in the omega-3 and omega-6 fatty acid status that is not solely attributable to breastfeeding exposure. The use of a binary breastfeeding variable (yes/no) does not capture important aspects such as exclusivity, duration, or feeding frequency. While this simplified classification allowed for maximal inclusion and statistical power, it may not fully reflect the complexity of infant feeding practices. This approach is consistent with WHO guidelines for population-based studies where detailed feeding data are unavailable [79, 80]. Prior studies have demonstrated that any breastfeeding exposure, regardless of duration or exclusivity, is associated with beneficial effects on infant metabolic, immune, and neurodevelopmental outcomes [81, 82].

We considered several pre-specified key confounders in these analyses and have considered additional adjustment for postnatal weight in sensitivity analyses, but unmeasured confounding may contribute to the beneficial effects of breastfeeding. We have considered infections here as the total number of infections, which we have previously shown is associated with inflammation and metabolic differences in infancy in this cohort [24]. Future work investigating clinical types and severity of infections may better elucidate pathways underpinning the effects of breastfeeding and highlight additional intervention targets. The Barwon Infant Study has relatively low ethnic minority representation, reducing generalisability of the findings.

## Conclusions

Breastfeeding up to 12 months of age is associated with reduced infection burden and inflammation, with evidence for breastfeeding-associated differences in metabolomic biomarkers and lipids, particularly ether lipids and plasmalogens, mediating much of these beneficial effects. There is also cross-sectional evidence for bidirectional mediation, with reduced infection burden and inflammation potentially mediating the effects of breastfeeding on some components of metabolic health. These findings may inform targets to improve early life metabolic health and inflammation burden, particularly in infants who are not breastfed. Future work should explore the functional role of the specific lipid pathways contributing to differential outcomes in breastfed and formula-fed infants. Understanding the metabolic pathways involved in the development of chronic inflammation in early life could inform the development of early prevention strategies to reduce the longer-term disease risks.

## Supplementary Information

Additional file 1: Tables S1–S45. Figures S1 and S2. Figure S1. Flowchart of Barwon Infant Study participants included in this study at the 6-month and 12-month timepoints. Figure S2. Comparison of proportion mediation by metabolomic biomarkers/lipids vs proportion mediation by GlycA.

## Data Availability

Access to BIS data including all data used in this paper may be requested through the BIS Steering Committee by contacting the corresponding author. Requests to access cohort data are considered on scientific and ethical grounds and, if approved, provided under collaborative research agreements. Deidentified cohort data can be provided in Stata or CSV format. All statistical methods used are referenced within the methods section. Additional project information, including cohort data description and access procedures, is available at the project’s website [https://www.barwoninfantstudy.org.au](https://www.barwoninfantstudy.org.au).

## Abbreviations

BIS: Barwon Infant Study
ACME: Average causal mediation effect
ADE: Average direct effect
BMI: Body mass index
C: Cholesterol
CE: Cholesteryl esters
CVD: Cardiovascular disease
DG: Diacylglycerol
DHA: Docosahexaenoic acid
FA: Fatty acids
FC: Free cholesterol
FDR: False discovery rate
GlycA: Glycoprotein acetyls
GP: General practitioner
HDL: High-density lipoprotein
HexCer: Hexosylceramide
HREC: Human research ethics committee
hsCRP: High-sensitivity C-reactive protein
IQR: Interquartile range
L: Large
LC/MS: Liquid chromatography/mass spectrometry
LDL: Low-density lipoprotein
M: Medium
MRM: Multiple reaction monitoring
MUFA: Monounsaturated fatty acids
NCD: Non-communicable disease
NMR: Nuclear magnetic resonance
PC: Phosphatidylcholine
PC (O): Alkyl phosphatidylcholine
PC (P): Alkenyl phosphatidylcholine
PE: Phosphatidylethanolamine
PE (O): Alkyl phosphatidylethanolamine
PE (P): Alkenyl phosphatidylethanolamine
PG: Phosphatidylglycerol
PL: Phospholipid
PUFA: Polyunsaturated fatty acids
ROS: Reactive oxygen species
SEIFA: Socio-Economic Indexes for Areas
SFA: Saturated fatty acids
SM: Sphingomyelin
TG: Triacylglycerol
TG (O): Alkyl-diacylglycerol
VLDL: Very low-density lipoprotein
WHO: World Health Organisation
XXL: Extremely large
